# Genetic Interaction of *Aspergillus nidulans galR*, *xlnR* and *araR* in Regulating D-Galactose and L-Arabinose Release and Catabolism Gene Expression

**DOI:** 10.1371/journal.pone.0143200

**Published:** 2015-11-18

**Authors:** Joanna E. Kowalczyk, Birgit S. Gruben, Evy Battaglia, Ad Wiebenga, Eline Majoor, Ronald P. de Vries

**Affiliations:** 1 Fungal Physiology, CBS-KNAW Fungal Biodiversity Centre & Fungal Molecular Physiology, Utrecht University, Utrecht, the Netherlands; 2 Microbiology, Utrecht University, Utrecht, the Netherlands; University of Nebraska, UNITED STATES

## Abstract

In *Aspergillus nidulans*, the xylanolytic regulator XlnR and the arabinanolytic regulator AraR co-regulate pentose catabolism. In nature, the pentose sugars D-xylose and L-arabinose are both main building blocks of the polysaccharide arabinoxylan. In pectin and arabinogalactan, these two monosaccharides are found in combination with D-galactose. GalR, the regulator that responds to the presence of D-galactose, regulates the D-galactose catabolic pathway. In this study we investigated the possible interaction between XlnR, AraR and GalR in pentose and/or D-galactose catabolism in *A*. *nidulans*. Growth phenotypes and metabolic gene expression profiles were studied in single, double and triple disruptant *A*. *nidulans* strains of the genes encoding these paralogous transcription factors. Our results demonstrate that AraR and XlnR not only control pentose catabolic pathway genes, but also genes of the oxido-reductive D-galactose catabolic pathway. This suggests an interaction between three transcriptional regulators in D-galactose catabolism. Conversely, GalR is not involved in regulation of pentose catabolism, but controls only genes of the oxido-reductive D-galactose catabolic pathway.

## Introduction

Filamentous fungi are able to degrade structural plant cell wall polysaccharides (e.g. xylan and pectin) and catabolize the released monosaccharides [1]. The production of the enzymes needed for polysaccharide degradation is controlled by transcriptional regulators. Some of these regulators have been identified and characterized in *Aspergillus*, such as the transcriptional activators AraR, GalR and XlnR [2–4]. These regulators are most likely induced by L-arabinose/L-arabitol, D-galactose and D-xylose, respectively, and also control the metabolic conversion of these monosaccharides. This provides the fungus with a quick and controllable system that responds to the polymers present in the environment. Notably, these monosaccharides often co-occur in nature, in particular in plant biomass. Xylan, the most abundant hemicellulose in hardwoods and cereals, has a backbone of β-1, 4-linked D-xylose units that can be branched with L-arabinose and D-galactose, but also acetyl, feruloyl, *p*-coumaric and D-glucuronic acid [1]. Arabinogalactan, commonly found in softwoods like larch, is made of branched D-galactose units with L-arabinose side chains. Xyloglucan is a polysaccharide present in all terrestrial plants [5]. It consists of β-1, 4-linked D-glucose chains that can be, depending on the type, substituted with D-xylose, L-arabinose and D-galactose. The primary cell wall of terrestrial plants is also rich in pectin [6]. Pectin is a complex polymer build from four structural elements: homogalacturonan, xylogalacturonan, and rhamnogalacturonan (I and II). Rhamnogalacturan-I part has a backbone of alternating D-galacturonic acid and L-rhamnose residues, with side chains that contains significant amounts of L-arabinose and/or D-galactose. The xylogalacturonan part of pectin consists of a D-galacturonic acid backbone with β-linked residues of D-xylose. These common plant polysaccharides provide a simultaneous source of D-xylose, L-arabinose and D-galactose for the fungus. Therefore, the intracellular pathways that convert them could also work simultaneously.

**Fig 1:**
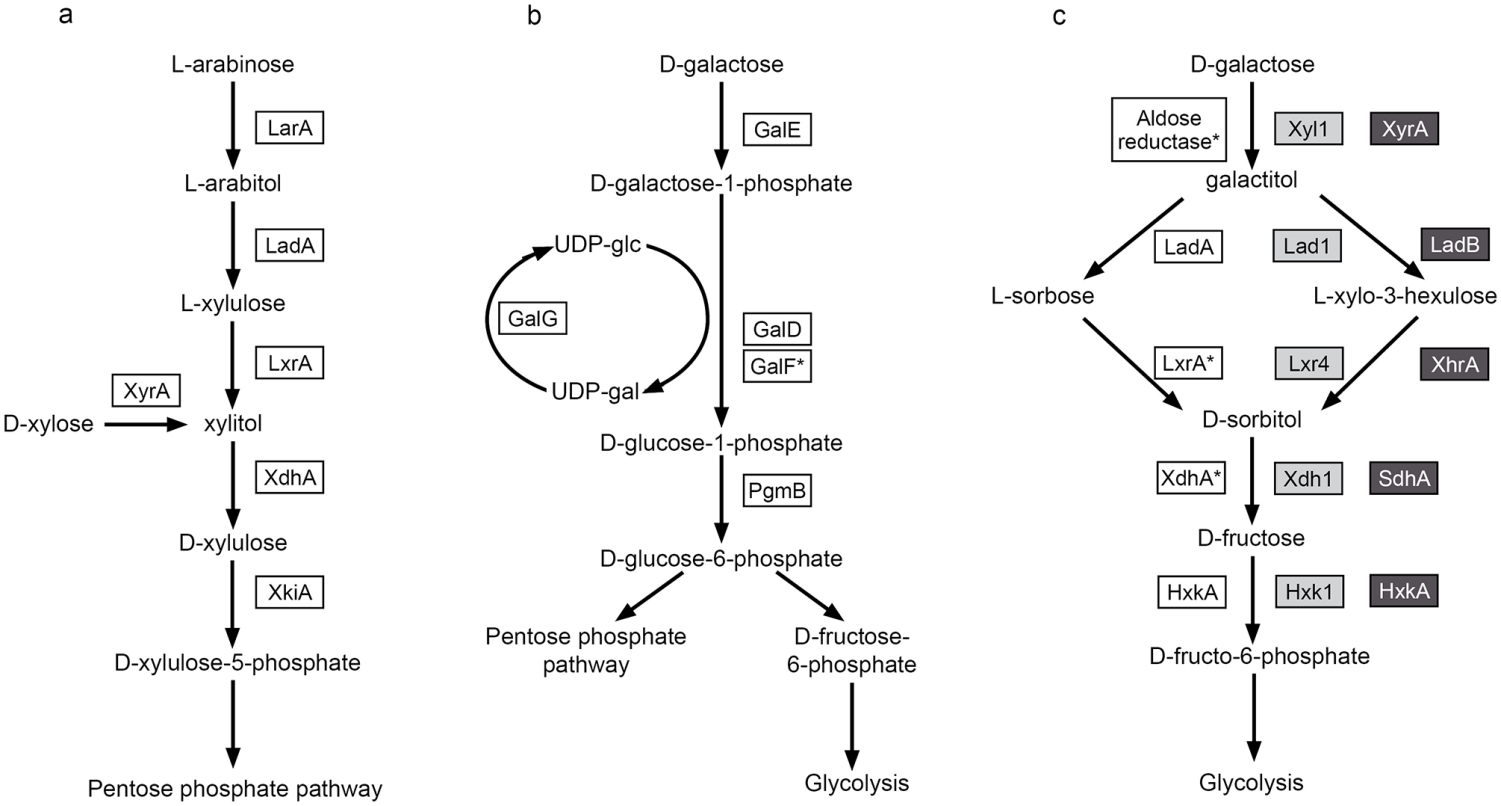
The pentose catabolic pathway (a), the Leloir pathway (b) and the oxido-reductive pathway (c) that convert arabinose, xylose and galactose. The enzymes that catalyse particular metabolic steps in *A*. *nidulans* (white boxes), *T*. *reesei* (light grey boxes) and *A*. *niger* (dark grey boxes) are indicated. In the oxido-reductive pathway in *A*. *nidulans* the product of galactitol oxydation is most likely L-sorbose, while in *A*. *niger* and *T*. *reesei* this product was characterised as L-xylo-3-hexulose [13,14]. Unidentified or unconfirmed enzymes are marked with a star. Gene numbers of *A*. *nidulans* enzymes: LarA = AN7193, LadA = AN0942, LxrA = AN10169, XdhA = AN9064, XyrA = AN0423, XkiA = AN8790, GalE = AN4957.3, GalD = AN6182.3, GalF = 9148.3, GalG = AN4727.3, PgmB = AN2867.3, XxkA = AN7459. Gene numbers of *A*. *niger* enzymes: XyrA = An01g03740, LadB = An16g01710, XhrA = An16g01650, SdhA = An07g01290, HxkA = An02g14380.

The pentose catabolic pathway (PCP) converts both L-arabinose and D-xylose to D-xylulose-5-phosphate, which then enters the pentose phosphate pathway (PPP) [7]. In L-arabinose catabolism, L-arabinose is converted to L-arabitol, L-xylulose and xylitol by L-arabinose reductase (LarA), L-arabitol-4-dehydrogenase (LadA) and L-xylulose reductase (LxrA), respectively (Fig 1a) [8–10]. D-xylose is converted to xylitol by L-xylose reductase (XyrA) [11]. Xylitol is then converted to D-xylulose and D-xylulose-5-phosphate by xylitol dehydrogenase (XdhA) and D-xylulose kinase (XkiA), respectively [9,12]. AraR is the main regulator of the PCP genes, with the exception of XyrA which is under control of XlnR [3]. AraR is present only in Aspergilli and other species from the order Eurotiales while XlnR is present in most Ascomycetes [3].

Filamentous fungi use several pathways for D-galactose catabolism. The best studied pathway is the Leloir pathway, which is present in prokaryotic and eukaryotic organisms [14]. In *A*. *nidulans* the first step of the Leloir pathway is a conversion of D-galactose to D-galactose-1-phosphate catalyzed by galactokinase (GalE) (Fig 1b). The second step is catalyzed by D-galactose-1-phosphate-uridylotransferase (GalD) and generates two products, UDP-galactose and D-glucose-1-phosphate. UDP-galactose can then be re-cycled to UDP-glucose by UDP-galactose-4-epimerase (GalG) and D-glucose-1-phosphate is converted to D-glucose-6-phosphate by phosphoglucomutase (PgmB) [14–16].

*A*. *nidulans* also possesses an oxido-reductive D-galactose catabolic pathway that converts D-galactose to galactitol, which is then converted to L-sorbose [17]. L-sorbose is reduced to D-sorbitol that in turn is converted to D-fructose and D-fructose-1-phosphate (Fig 1c). Not all enzymes that catalyze particular steps in *A*. *nidulans* have been identified but similarity to *T*. *reesei* was suggested [4,17]. In *T*. *reesei* the oxido-reductive D-galactose conversion involves three enzymes of the PCP: Xyl1, Lad1 and Xdh1 (Fig 1c). Aldose reductase (Xyl1) is the major enzyme that catalyzes the reduction of D-galactose as well as reduction of L-arabinose and D-xylose [18]. L-arabitol dehydrogenase (Lad1) catalyzes conversion of both L-arabitol to L-xylulose [19] and galactitol to L-xylo-3-hexulose [20] and the corresponding gene is upregulated in the presence of L-arabinose and D-galactose [20]. Xylitol dehydrogenase (Xdh1) was proposed to catalyze oxidation of both xylitol and sorbitol [13].

Distinct enzymes are involved in some enzymatic steps of the oxido-reductive D-galactose catabolic pathway in *A*. *niger*. The *ladA* gene of *A*. *niger*, homologous to *T*. *reesei lad1*, was induced on L-arabinose but not on D-galactose or galactitol [21]. *A*. *niger* has a separate galactitol dehydrogenase gene, *ladB*, catalyzing the conversion of galactitol to L-xylo-3-hexulose [21]. This gene is also present in *A*. *nidulans*. The next step, reduction of L-xylo-3-hexulose to D-sorbitol is catalyzed by L-xylo-3-hexulose reductase, XhrA, not by LxrA in *A*. *niger* [13]. Conversion of D-sorbitol to D-fructose is catalyzed by a sorbitol dehydrogenase (SdhA) [22] (Fig 1c). The first step of this conversion pathway, D-galactose reduction, also involves D-xylose reductase (XyrA) from PCP, as was shown in *T*. *reesei* [21].

The regulation of D-galactose catabolic pathways in *A*. *nidulans* remains unclear, although some interesting indications for interactions between the D-galactose-responsive regulator, GalR, and arabinanolytic regulator, AraR, have been suggested. In the previous study [4], a model of D-galactose catabolism in *A*. *nidulans* and *T*. *reesei* was presented in which LadA (in principal involved in PCP) was predicted to catalyze also D-galactitol conversion in D-galactose oxido-reductive pathway. Moreover, the expression of *ladA* was reported to be controlled by GalR on D-galactose [4] while expression of this gene on L-arabinose is known to be under control of AraR [3]. In *A*. *nidulans* two transcriptional factors (TFs), GalR and GalX, which respond to the presence of D-galactose were identified [4]. GalX is present in most *Aspergillus* species, while GalR was described as specific for *A*. *nidulans* [4]. The deletion of *galR* in *A*. *nidulans* or *galX* in *A*. *nidulans* and *A*. *niger* causes reduced growth on D-galactose and galactitol and several genes of oxido-reductive and Leloir pathways seem to be affected by this mutation [4,23].

In this study, we investigated the genetic interaction between XlnR, AraR and GalR in detail by studying the phenotype of single, double and triple disruptants of genes encoding these regulators in *A*. *nidulans*. Moreover, we present the influence of those mutations on the expression of genes from pentose and D-galactose catabolism.

## Material and Methods

### Strains, culture conditions and media

*A*. *nidulans* strains (Table 1) were grown at 37°C using minimal medium (MM) or complete medium (CM) [24]. Plates of these media contained 1.5% agar. Spore plates contained CM + 2% glucose, while plates used in growth experiments contained MM + 25 mM monosaccharide or 1% polysaccharide. The pH of the medium containing apple pectin was adjusted to 6. The supplements arginine or uridine were added (0.2 g L^-1^ and 1.2 g L^-1^, respectively) when required. Liquid cultures were grown on a rotary shaker at 250 rpm. Pre-cultures for RNA isolation were grown in 1 L Erlenmeyer flasks containing 250 ml CM with 2% D-fructose. After 16h of incubation, the mycelium was harvested, washed with MM and transferred to 250 ml Erlenmeyer flasks containing 50 ml MM + 25 mM carbon source (D-fructose, D-xylose, L-arabinose or D-galactose). After 2 h of incubation, the mycelium was harvested by vacuum filtration, dried between tissue paper and frozen in liquid nitrogen. New mutant strains were deposited at CBS Fungal Biodiversity Centre with strain numbers indicated in Table 1.

**Table 1:** *A*. *nidulans* strains used in this study.

Strain	Code	Strain number	Genotype	Reference
AN031	Ref	CBS 129193	*pyrG8*, *argB2*	Battaglia, 2011a
Δ*araR*	ΔA	CBS 129196	*pyrG89*, *argB2*, *ΔaraR*::*pyrG*+	Battaglia, 2011a
Δ*xlnR*	ΔX	CBS 129195	*pyrG89*, *argB2*, *ΔxlnR*::*argB*+	Battaglia, 2011a
Δ*galR*	ΔG	CBS 138911	*pyrG89*, *argB2*, *ΔgalR*::*pyrG*+	This study
Δ*xlnR*/Δ*araR*	ΔXΔA	CBS 129197	*pyrG89*, *argB2*, *ΔaraR*::*pyrG+*, *ΔxlnR*::*argB*+	Battaglia, 2011a
Δ*xlnR*/Δ*galR*	ΔXΔG	CBS 138912	*pyrG89*, *argB2*, *ΔxlnR*::*argB+*, *ΔgalR*::*pyrG+*	This study
Δ*araR*/Δ*galR*	ΔAΔG	CBS 138910	*pyrG89*, *argB2*, *ΔaraR*::*pyrG*+, *ΔgalR*::*pyrG*+	This study
Δ*xlnR*/Δ*araR*/Δ*galR*	ΔΔΔ	CBS 138909	*pyrG89*, *argB2*, *ΔaraR*::*pyrG*, *ΔxlnR*::argB+, *ΔgalR*::*pyrG*+	This study

### Sexual crosses

Sexual crosses between Δ*xlnR*, Δ*araR* and Δ*galR* strains were performed as described previously [25]. Strains were screened by selecting for poor growth on L-arabinose, D-xylose and/or D-galactose. Absence of the regulatory genes was verified by Southern blot analysis.

### Molecular biology methods

Molecular biology methods were performed according to standard procedures [26], unless stated otherwise. For gDNA and RNA isolation frozen mycelium was ground using a TissueLyser II (QIAgen). RNA was extracted using TRIzol reagent (Invitrogen) [27] and purified with NucleoSpin RNA II Clean-up kit (Macherey-Nagel) with DNase treatment. The RNA quantity of the samples was checked with a NanoDrop-1000 spectrophotometer and the quality by RNA gel electrophoresis. Total RNA in amount of 2.5 μg was reverse transcribed using the ThermoScript^™^ RT-PCR System (Invitrogen, Carlsbad, USA) and obtained cDNA was diluted 100x and used in the qRT-PCR reaction.

### Gene expression assays

Gene expression was assayed by quantitative real-time PCR (qRT-PCR; Applied Biosystems 7500 Real-time PCR system) using ABI Fast SYBR Master Mix (Applied Biosystems, Foster City, CA, USA). The primers were designed using PrimerExpress^®^ 3.0 software (Applied Biosystems, Foster City, CA, USA) according to the supplier’s instructions (Table 2) and validated experimentally. Optimal primer concentrations were determined by analysis of dissociation curves for amplicons generated by combinations of 50nM, 300nM and 900nM (final concentrations) primer per pair. The efficiency of optimal primer pairs was assessed using the correlation between the cycle threshold value (Ct) and the logarithm of *A*. *niger* gDNA dilutions (10ng to 1pg). The amplification efficiency for all the primer couples was estimated at 91–101%. The qRT-PCR reaction contained 10μl ABI Fast SYBR Master Mix, 2μl forward and reverse primer (at optimal concentration) and 2μl of cDNA in the final volume of 20μl. The cycling conditions were 95°C for 20sec, followed by 40 cycles of 95°C for 3sec and 60°C for 30sec. A dissociation curve was generated to confirm that single product was amplified. Amplification quality was verified by using No Template Control (NTC), which contained 2μl of water instead of cDNA. Expression levels were normalized against β-tubulin (*tubC*) as a physiological reference and calculated according to the relative quantification 2^-ΔCt^ method [28]. Two biological and three technical replicates were analyzed. GraphPad Prism 6 (GraphPad Software Inc., La Jolla, CA, USA) was used to calculate the mean, standard deviation and significance of the samples. Statistical significance was determined using multiple unpaired two-tailed *t*-tests and corrected with Holm-Sidak method, with *P*-value <0.05.

**Table 2:** Primers used in this study to generate the gene fragments for qRT-PCR analysis (a) and Southern Blot (b).

Gene	Gene number	Forward	Reverse
**a)**			
*tubC*	AN6838	CGGAAACTGGCCGTCAATAT	CCACCACCGTATCCGACACT
*xlnR*	AN7610	CGCGCGTTCAATCACATC	CCGCTTGAGTGTTGTGAATACTAGA
*araR*	AN0388	GTCCGGCACTGCTTTCGA	GCAACGAAAACGCCCTATCA
*galR*	AN10550	TCGCTTACACTGGACCTAATGAGA	GCGTGGCCGTTTTTATCAA
*larA*	AN7193	GGAGGCCACTCGGTCATTC	TGGAAGTTCTCGGCAATGC
*ladA*	AN0942	ATCCGACGTGCACTTTTGG	TCCCCCGTGACGATCATG
*lxrA*	AN10169	TGGCCGCATAGTGAACATCTC	GCGCTTTGCCCGATGA
*xdhA*	AN9064	GTGCTGATGTCGCGATTGAT	CATGGATACCCGTGTGAACTGA
*xkiA*	AN8790	AACAGACCCGCCGATGAAG	GAGACGGAGAGAGAGCATTTGG
*xyrA*	AN0423	ACTCGTCGTTTGGGCCTTT	GCTCCTTCCGCCTGTTTCA
*galD*	AN6182	CCTCGCCAAACCAAATGAA	TGCCGCGCCCTTGTT
*galE*	AN4957	CAAGGCTGCGGAGGAAATC	CAGCTTTGTTGGAGGTAACGAA
*galF*	AN9148	CCTGGGCGCTGTTGTTG	CAGCCTTGGTGTCGATCATG
*ladB*	AN4336	CGGCTCAGACGTGCATTTC	CCCCGTCACGACCATAGG
*sdhA*	AN2666	ACCCACGAATTTTCTTTCTCC	CGACTGGCGACATTATGG
*hxkA*	AN7459	TGGACCAAGGGTTTCGACAT	CTCAAGAGGCGGGACTACGT
**b)**			
*xlnR*	AN7610	GCATCTTGGCGATGGGATC	TGGATTTCCGAGGCAGAGC
*araR*	AN0388	ACATTGGGGAATCCACATCC	GGTGACCAGGATTTCGTGG
*galR*	AN10550	TTGGACACAAGCTTTGACCG	TCGAAATCCAGGAAGATGGG

### Genomic cluster comparison

Synteny of *galX* and *galR* genes in the genome of *A*. *nidulans*, *A*. *versicolor*, *A*. *sydowii*, *A*. *fumigatus A1163*, *A*. *fumigatus Af293*, *A*. *flavus*, *A*. *oryzae*, *A*. *niger CBS 513*.*88*, *A*. *niger ATCC 1015*, *A*. *acidus*, *A*. *kawachii*, *A*. *tubigensis*, *A*. *brasiliensis*, *A*. *carbonarus*, *A*. *wentii* and *A*. *glaucus* was analyzed using the Sybil algorithm [29] at the Aspergillus Genome Database (AspGD; http://www.aspergillusgenome.org).

## Results and Discussion

**Fig 2:**
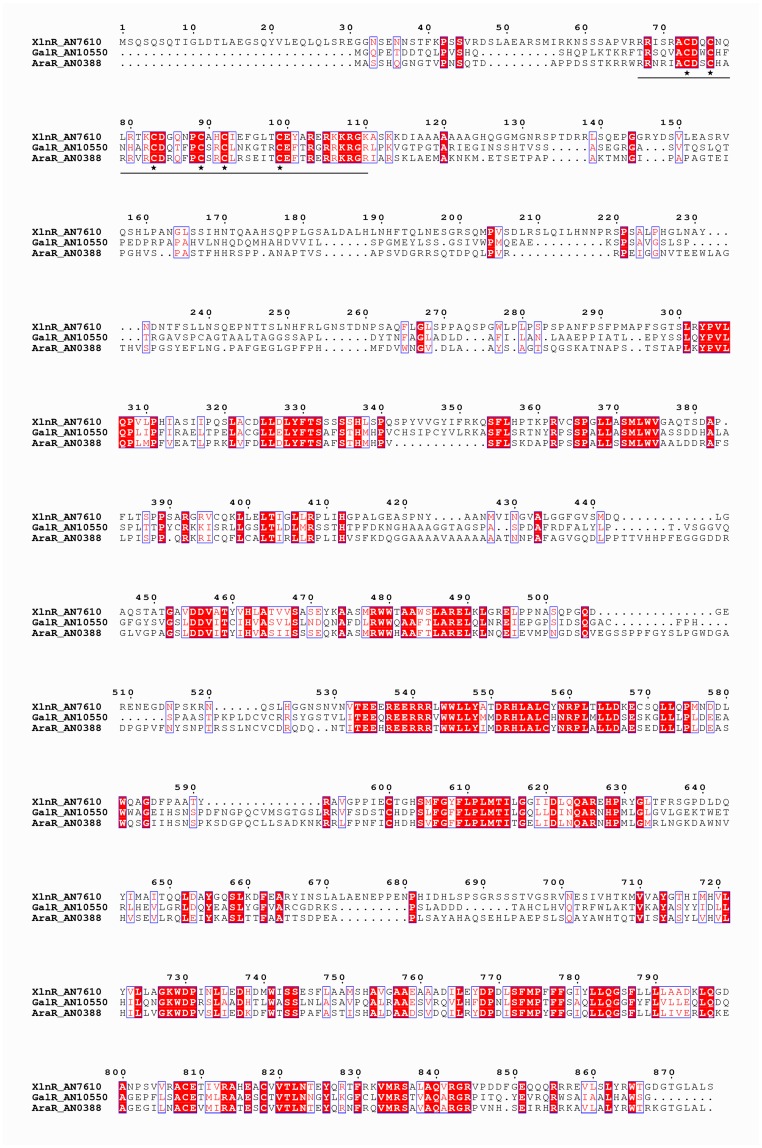
Amino acid sequence alignment of *A*. *nidulans* GalR, AraR and XlnR. The alignment was performed using Clustal Omega (http://www.ebi.ac.uk/Tools/msa/clustalo) [31] and visualized using Easy Sequencing in PostScript (http://espript.ibcp.fr/ESPript/ESPript/index.php) [32]. Conserved regions are marked by shaded boxes and similar regions by unshaded boxes. The Zn_2_Cys_6_ binuclear DNA binding domain is underlined and the six conserved cysteine residues are indicated by stars.

**Fig 3:**
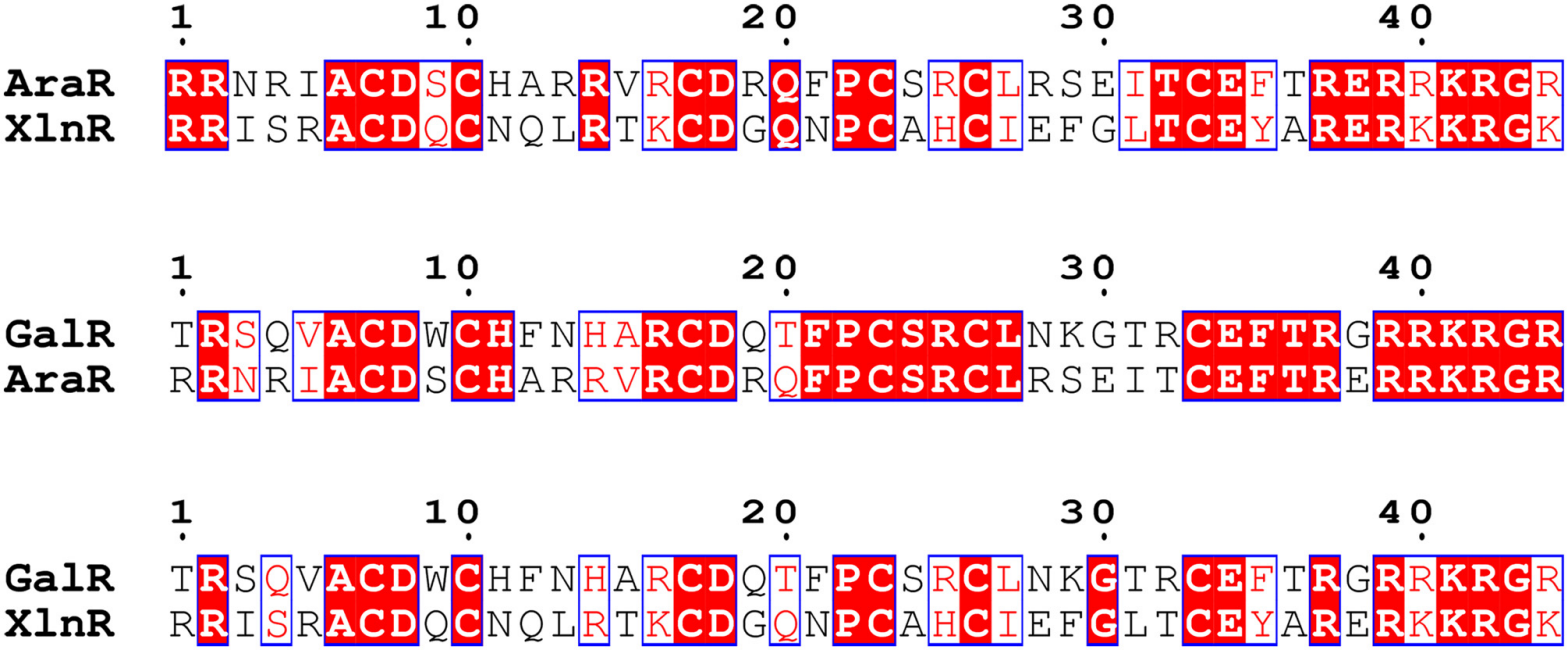
Pairwise amino acid sequence alignment within the DNA binding domain of GalR, AraR and XlnR in *A*. *nidulans*. The alignment was performed using Clustal Omega (http://www.ebi.ac.uk/Tools/msa/clustalo) [31] and visualized using Easy Sequencing in PostScript (http://espript.ibcp.fr/ESPript/ESPript/index.php) [32]. Conserved regions are marked by shaded boxes and similar regions by unshaded boxes.

In *A*. *nidulans*, two transcription factors (TFs) co-regulate genes of the pentose catabolic pathway (XlnR and AraR) and two TFs co-regulate genes of the galactose conversion pathways (GalR and GalX) [3,4]. In this study we investigated possible genetic interaction between three paralogous sugar-specific TFs (XlnR, AraR and GalR) in *A*. *nidulans*. The full length protein sequences of XlnR, AraR and GalR share more than 30% amino acid sequence identity (Fig 2) and are relatively conserved in both the DNA binding and activation domains [3,4]. Within the Zn_2_Cys_6_ binuclear DNA binding domain, AraR shares 51% and 63% sequence identity with XlnR and GalR, respectively (Fig 3, Table 3). Detailed protein alignments of TF pairs can be found in the supplementary data (S1 Fig). Co-involvement of AraR and XlnR in regulation of the PCP and PPP has been described [3,30], while the genetic interactions between AraR and XlnR was never addressed before. The higher level of sequence conservation in the DNA binding domains of AraR and GalR suggest they may also co-regulate the same target genes. Less conservation was observed within the DNA binding domain of GalR and XlnR (Table 3; 44% identity), which is similar to the conservation degree between the non-paralogous TFs XlnR and InuR (43% identity). Despite the lower degree of conservation between these two paralogs, we cannot exclude possible genetic interaction between *galR* and *xlnR*. In this study we will elucidate co-regulation between all three TFs in the PCP and galactose conversion pathways. We constructed double and triple regulator *A*. *nidulans* mutants to study their growth phenotype on different carbon sources and expression levels of metabolic genes.

**Table 3:** Amino acid sequence identity (%) within the Zn_2_Cys_6_ binuclear DNA binding domain of GalR, AraR and XlnR in *A*. *nidulans*. InuR is an example of non-paralogous TF.

	GalR (AN10550)	AraR (AN0388)	XlnR (AN7610)	InuR (AN3835)
GalR (AN10550)	-			
AraR (AN0388)	63% (27/43)	-		
XlnR (AN7610)	44% (17/39)	51% (20/39)	-	
InuR (AN3835)	29% (11/38)	39% (15/38)	43% (17/40)	-

**Fig 4:**
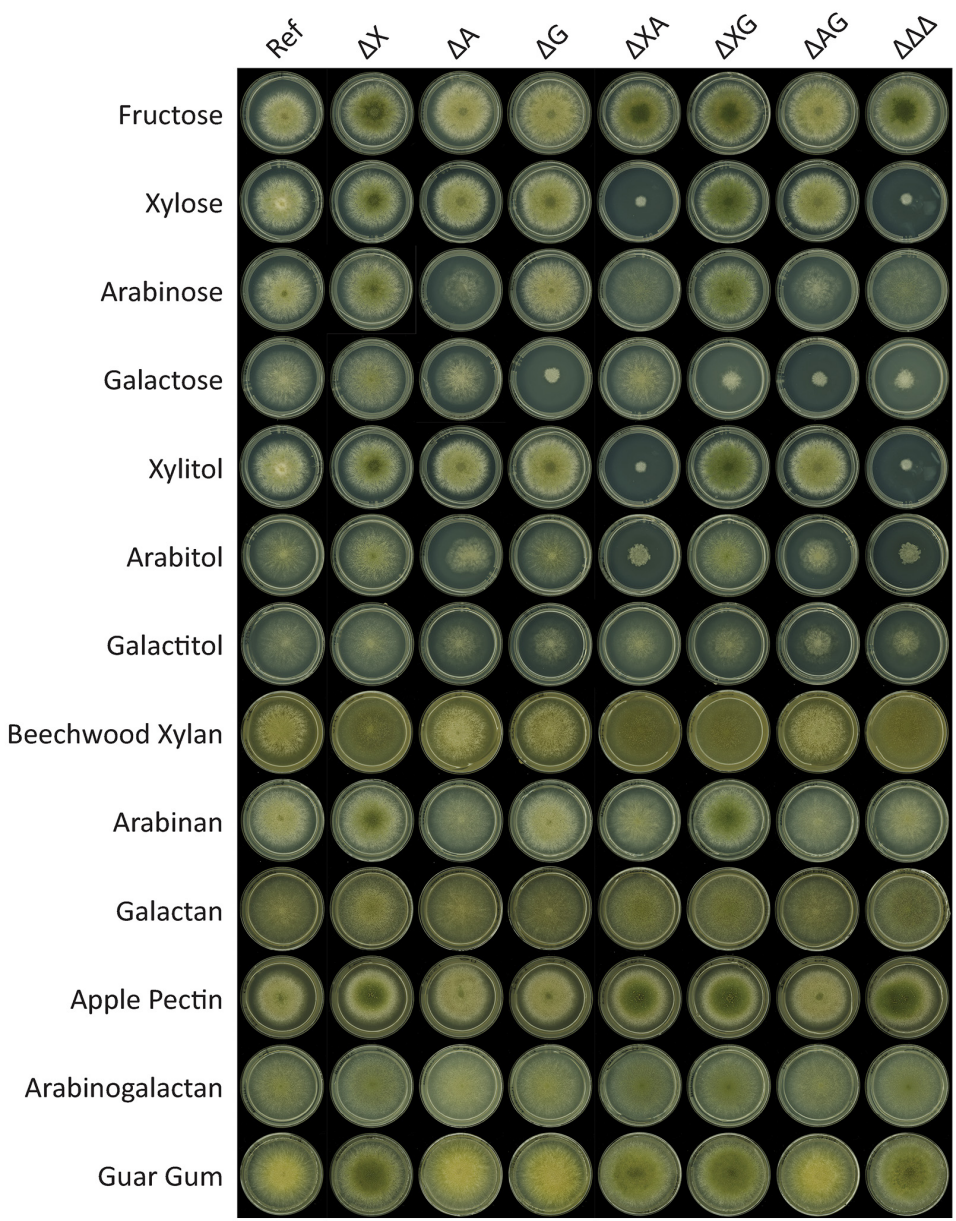
Growth profiling of an *A*. *nidulans* reference strain and single, double and triple disruptants of *araR*, *xlnR*, and *galR* on medium containing different carbon sources. Strain codes are described in Table 1. Strains were grown for 3 days at 37°C.

**Fig 5:**
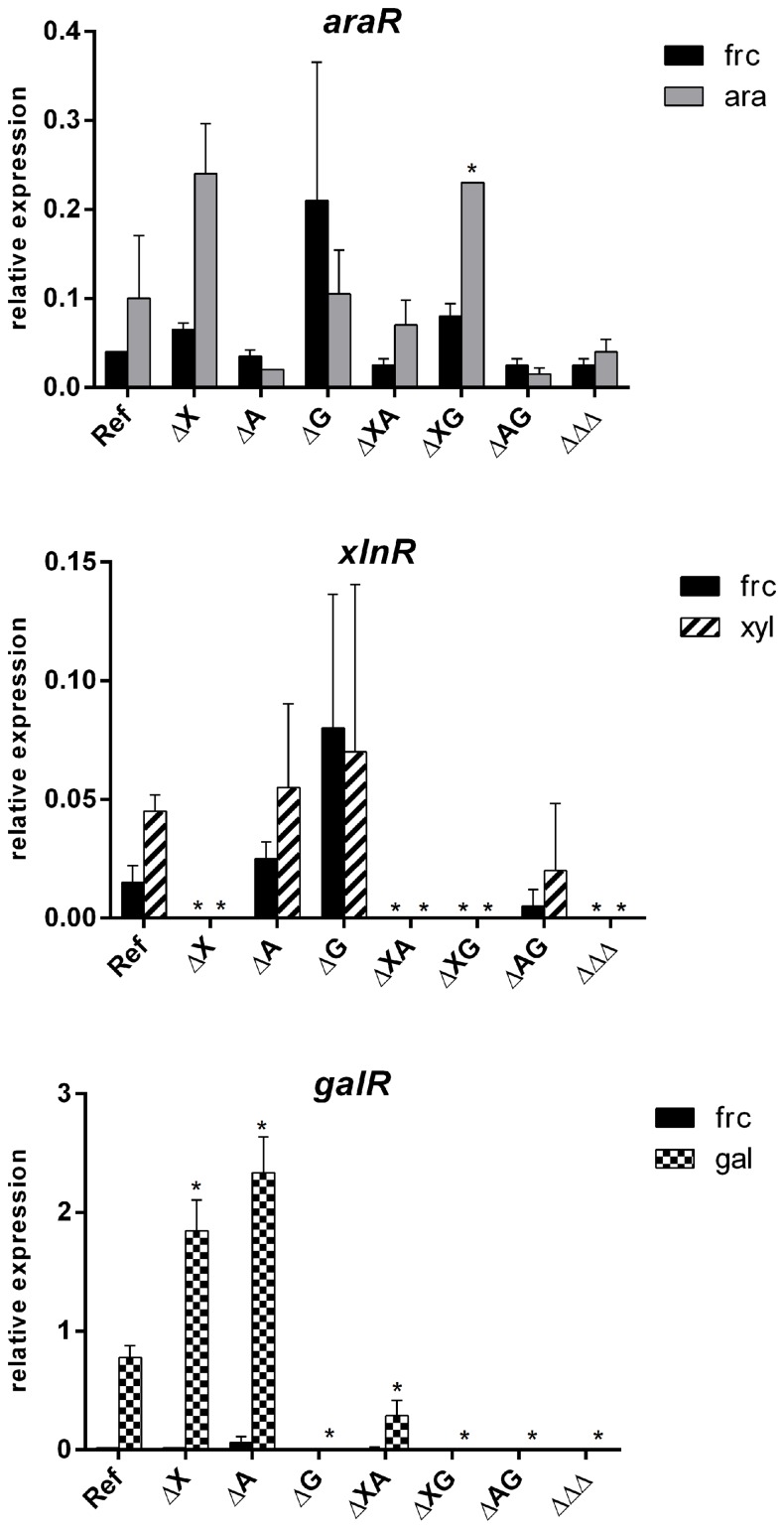
Expression levels of *araR*, *xlnR* and *galR* genes in ΔAraR, ΔXlnR and ΔGalR mutants in *A*. *nidulans*. The expression was measured in the reference and single, double and triple regulatory mutant strains of *A*. *nidulans* after 2h of growth on D-fructose (black bars), L-arabinose (grey bars), D-xylose (striped bars) and D-galactose (checkered bars). The columns represent the mean and error bars represent standard deviation between biological replicates. Significant change in expression between the reference strain and the mutant was marked by the asterisk.

Growth of the *A*. *nidulans* reference strain, and single (ΔX, ΔA and ΔG), double (ΔXΔA, ΔXΔG, ΔAΔG), and triple (ΔΔΔ) disruptants (see Table 1 for strain designations) was compared on different sugars (mono- and polysaccharides) with fructose as a reference carbon source (Fig 4). The influence of the nitrogen source was tested by growing the strains in the presence of two different nitrogen sources, ammonium and nitrate respectively, but no differences in growth on the carbon sources were observed (data not shown). Expression of orthologous genes involved in PCP and oxido-reductive D-galactose catabolic pathway in *T*. *reesei* (*larA*, *ladA*, *lxrA*, *xyrA*, *xdhA*, *xkiA*), and from the oxido-reductive D-galactose pathway (*ladB*, *sdhA*, *hxkA*) and Leloir pathway (*galE*, *galD*, *galF*) in *A*. *niger* was analyzed by qRT-PCR after growth in media containing D-fructose, D-xylose, L-arabinose or D-galactose as a carbon source. Expression of *araR*, *xlnR* and *galR* was measured to confirm the deletion of the regulator in the respective strains. In addition, this was used to determine how the regulator encoding genes respond to the disruption of each other (Fig 5).

**Fig 6:**
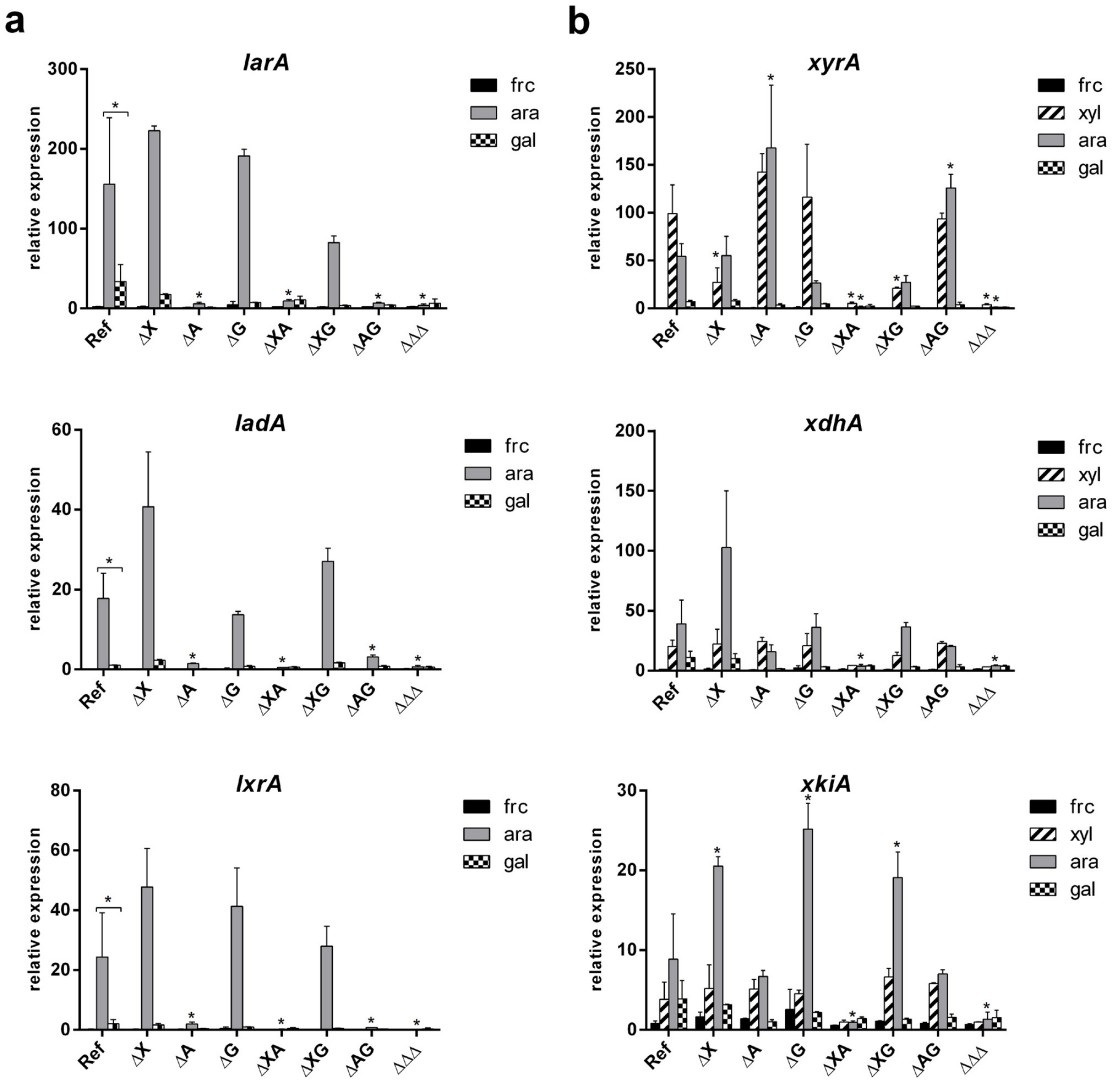
Expression levels of six genes encoding enzymes involved in the PCP in *A*. *nidulans*. The expression of *larA*, *ladA*, *lxrA* (**a**) and *xyrA*, *xdhA*, *xki* (**b**) was measured in the reference and regulatory mutant strains of *A*. *nidulans* after 2h of growth on D-fructose (black bars), L-arabinose (grey bars), D-xylose (striped bars) and D-galactose (checkered bars). The columns represent the mean and error bars represent standard deviation between biological replicates. Significant change in expression between the reference strain and the mutant was marked by the asterisk. The asterisk and the brace represent the significance between two indicated conditions.

Growth of *A*. *nidulans* on L-arabinose and L-arabitol was reduced in all strains in which *araR* is absent (Fig 4). This correlates with the expression results which showed that the first three genes involved in PCP (*larA*, *ladA*, *lxrA*) are under control of AraR as they are significantly down regulated in all *ΔaraR* strains on L-arabinose (Fig 6a). The expression of those genes on D-fructose is absent and it is induced on L-arabinose (Fig 6a). This confirms the previously described role of AraR as the major regulator of the PCP genes, and in particular its influence on enzymatic steps that are required for L-arabinose conversion [3]. The growth on arabinan was decreased for the strains in which *araR* was absent (Fig 4). This confirms that the role of AraR extends to regulation of expression of genes encoding extracellular enzymes. This phenomenon was already described in *A*. *niger*, in which expression of the α-arabinofuranosidase encoding genes *abfA* and *abfB* are under regulatory control of AraR [3].

Growth of *A*. *nidulans* on D-xylose and xylitol was only affected when both *xlnR* and *araR* are deleted (Fig 4). The last two enzymes of the PCP (*xdhA*, *xkiA*) and D-xylose reductase (*xyrA*) are co-regulated by XlnR and AraR and only the Δ*xlnR* Δ*araR* double mutant showed significantly reduced expression on L-arabinose (Fig 6b). Reduced expression of *xyrA* gene in ΔAΔX interrupted conversion of D-xylose via the pentose catabolic pathway and caused the observed growth reduction. Similarly, reduced expression of *xdhA* gene in ΔAΔX caused reduced growth on xylitol (Fig 4). Interesting expression patterns of those XlnR and AraR co-regulated genes were observed in single mutants. The *xdhA* and *xkiA* genes were highly expressed in the absence of XlnR and not reduced in the absence of AraR. The *xyrA* gene shows the opposite pattern, it is not reduced in the absence of XlnR and highly expressed in the absence of AraR. These observations indicate which TF is the predominate activator of the pentose catabolic pathway gene (Table 4). All the genes involved in the PCP in *A*. *nidulans* showed low expression in triple and some double knockout strains on L-arabinose (Fig 6a and 6b). This observed low level of expression may be a basal constitutive expression. A similar low expression level was seen in the reference strain on D-fructose, which is not an inducer of the pentose catabolic pathway (Fig 6a and 6b). On beechwood xylan, reduction in colony density but not in the diameter was observed in all strains in which *xlnR* was absent (Fig 4). The growth of single, double and triple mutants of *xlnR*, *araR* and *galR* was not affected on galactan (galactose), apple pectin (D-galacturonic acid, rhamnose, arabinose, galactose, xylose), arabinogalactan (arabinose, galactose) and guar gum (mannose, galactose) (Fig 4). This demonstrated that the influence of those mutations is mainly on intracellular level.

**Table 4:** Detailed regulation of genes involved in the PCP in *A*. *nidulans*.

Gene number	Gene	Transcriptional activator
AN7193	*larA*	AraR alone
AN0942	*ladA*	AraR alone
AN10169	*lxrA*	AraR alone
AN0423	*xyrA*	AraR and XlnR, XlnR is predominate
AN9064	*xdhA*	AraR and XlnR, AraR is predominate
AN8790	*xkiA*	AraR and XlnR, AraR is predominate

It was previously suggested that the conversion of galactitol to L-sorbose could be catalyzed by L-arabitol dehydrogenase, LadA, which is normally involved in arabitol oxidation in *A*. *nidulans* [17]. The expression of *ladA* was reported to be controlled by GalR [4]. To test the hypothesis that *ladA* and the other enzymes of the PCP could be under regulation of GalR in *A*. *nidulans*, we analyzed the expression of *larA*, *ladA*, *lxrA*, *xdh*, *xkiA and xyrA* on both L-arabinose and D-galactose. The expression of *larA*, *ladA* and *lxrA* was significantly lower on D-galactose compared to L-arabinose in the reference strain (Fig 6a). Similar to previous results, the PCP genes are induced on L-arabinose compared to D-fructose. However, most of the PCP genes seem not to be induced on D-galactose and show only a basal level of expression. Moreover, deletion of *galR* did not reduce the expression of *larA*, *ladA*, *lxrA*, *xdhA*, *xkiA* and *xyrA* on D-galactose and L-arabinose (Fig 6a and 6b). Therefore, we conclude that GarR is not involved in regulation of expression of PCP genes on D-galactose and L-arabinose. In contrast, our data suggest that both AraR and XlnR are involved in regulation of D-galactose oxido-reductive catabolic pathway (see below).

**Fig 7:**
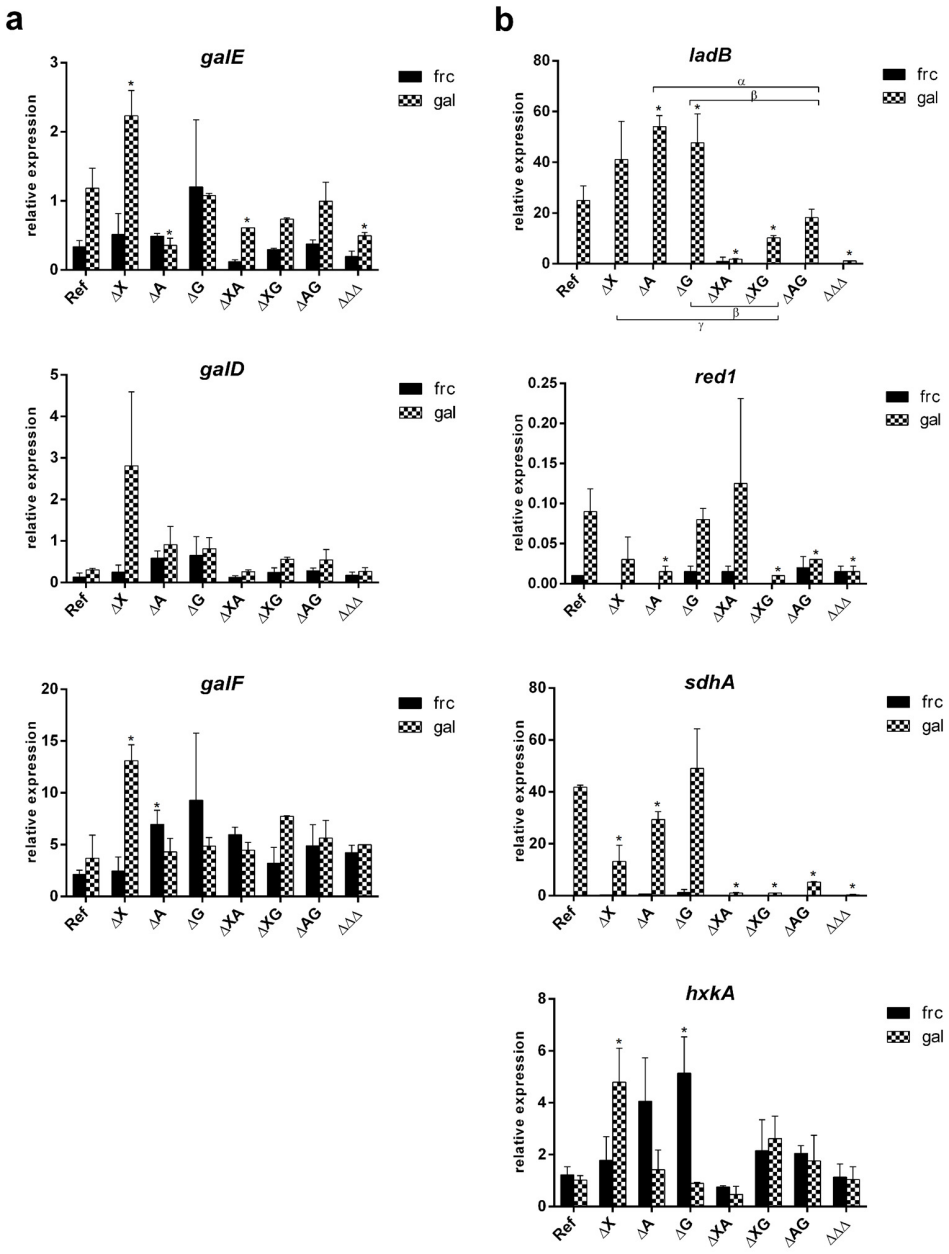
Expression of orthologous genes encoding enzymes involved in the Leloir (a) and the D-galactose oxido-reductive pathway (b) in *A*. *niger*. The expression was measured in the reference and regulatory mutant strains of *A*. *nidulans* after 2h of growth on D-fructose (black bars) and D-galactose (checkered bars). The columns represent the mean and error bars represent standard deviation between biological replicates. Significant change in expression between the reference strain and the mutant was marked by the asterisk. Significant changes in expression between single and double mutants were marked by Greek symbols and braces: an alpha (α) represents a significant change between ΔA and ΔAΔG, a beta (β) represents a significant change between ΔG and ΔXΔG/ΔAΔG, a gamma (γ) represents a significant change between ΔX and ΔXΔG.

Growth on D-galactose was reduced when GalR is absent, suggesting that GalR regulates the genes involved in D-galactose conversion (Fig 4) [4]. A previous study suggested that GalR is regulating the expression of galactokinase gene *galE* which converts D-galactose to D-galactose-1-phosphate in the Leloir pathway [4]. We compared expression levels of genes involved in the Leloir and the oxido-reductive D-galactose catabolic pathways in *galR* mutant and wild type grown of D-galactose as a carbon source. Expression of the Leloir pathway genes *galD*, *galE* and *galF* was not affected by the absence of GalR (Fig 7a) and therefore it cannot explain the observed growth reduction. In the alternative oxido-reductive D-galactose catabolic pathway D-galactose is converted to D-galactitol by a yet unknown aldose reductase. To explain the observed growth reduction in ΔG, we hypothesize that this unknown enzyme is regulated by GalR. It is worth to notice that the growth was highly reduced but not abolished in ΔG on D-galactose (Fig 4). The residual growth of ΔG could be caused by D-galactose catabolized through the unaffected Leloir pathway, but it also suggests that the oxido-reductive pathway is the preferred D-galactose conversion road in *A*. *nidulans*.

Growth on D-galactitol was slightly reduced when *araR* and/or *galR* were absent (Fig 4). Galactitol is an intermediate product in the oxido-reductive D-galactose pathway. Our findings negate the involvement of GalR-regulated *ladA* in conversion of galactitol. We deduced that *A*. *nidulans* similarly to *A*. *niger* has evolved distinct enzyme sets that catalyze the PCP and the D-galactose oxido-reductive pathway. The *ladB* gene has recently been characterized as a galactitol dehydrogenase encoding gene in *A*. *niger* [21]. In this study we tested the expression of the *ladB* ortholog in *A*. *nidulans*. Expression of *ladB* on D-galactose was high in the reference strain, significantly down regulated in ΔXΔG, and almost absent in ΔXΔA and ΔΔΔ (Fig 7b). This suggests that both XlnR and AraR (and to some point XlnR and GalR) are controlling *ladB*, but it does not explain what causes the growth reduction observed in single *araR* and *galR* mutants on galactitol. In the ΔXΔG double mutant, AraR is still present and probably compensates for the loss of the other two, which makes the change in expression less extreme (Fig 7b). Although there is no difference in *ladB* expression between the reference strain and the ΔAΔG on D-galactose, a strong reduction was observed when comparing *ladB* expression in the double mutant to the ΔA and ΔG single mutants. The fold change difference between ΔA and ΔAΔG is significant which means that the absence of GalR was (partially) responsible for this reduction (Fig 7b). A similar effect was observed when comparing ΔG and ΔAΔG. Altogether, the expression of *ladB* in the regulatory mutants illustrates high complexity of gene co-regulation. A previous study [4] suggested that *galR* and *ladB* are under control of GalX as the expression of those genes was lost in ΔGalX on D-galactose and galactitol. We cannot exclude that GalX, XlnR and AraR are regulating *ladB*. GalR was previously suggested to be uniquely present in *A*. *nidulans* [4], but the availability of additional *Aspergillus* genomes demonstrated that other members of the section nidulantes, *A*. *versicolor* and *A*. *sydowii*, contain an ortholog of this gene; however no orthologs were identified in any of the other sequenced *Aspergillus* species (S2 Fig).

*A*. *nidulans* does not possess an ortholog of *A*. *niger xhrA* and therefore we propose that another, yet undescribed reductase converts L-xylo-3-hexulose in this species. A previous study of the *A*. *niger* D-galactose oxido-reductive pathway [23] described a putative reductase encoding gene (*red1*), for which expression was strongly reduced (>30 fold change) in Δ*galX* compared to the wild type strain on D-galactose. We identified the ortholog of this gene (AN7914) in *A*. *nidulans* and analyzed its expression in the regulatory mutants. Similar to *ladB*, the expression of the *red1* gene was induced on D-galactose in the reference strain (Fig 7b). Moreover, this gene was significantly down regulated on D-galactose in the Δ*araR*, Δ*xlnR*Δ*galR* and Δ*araR*Δ*galR* double mutants and ΔΔΔ triple mutant (Fig 7b). We conclude that AN7914 is co-regulated by XlnR, GalR and AraR in *A*. *nidulans* and is a strong candidate to encode the not yet identified L-sorbose reductase. Further analysis, including *in vitro* characterization, needs to be performed to confirm its involvement in the D-galactose oxido-reductive pathway.

In the next step of the D-galactose oxido-reductive pathway in *A*. *niger*, D-sorbitol is converted to D-fructose by sorbitol dehydrogenase (SdhA) [22]. We identified the ortholog of *sdhA* (AN2666) in *A*. *nidulans* and analyzed its expression in the regulatory mutants. The *sdhA* gene was significantly down regulated on D-galactose in Δ*xlnR*, Δ*araR* and double disruptants of all three regulators tested (Fig 7b). The mutation in ΔXΔA and ΔXΔG had the strongest effect and abolished expression of *sdhA* gene (Fig 7b). Taking into account the expression levels of *ladB* and *sdhA* we propose that *A*. *nidulans*, similarly to *A*. *niger*, uses LadB and SdhA in the D-galactose oxido-reductive pathway. Both *ladB* and *sdhA* genes are highly induced by D-galactose and co-regulated by AraR, XlnR and GalR. Interestingly, interactions between those TFs might exceed beyond D-galactose metabolism. It was previously reported that β-galactosidase, an enzyme that removes β-linked D-galactose from polysaccharides, is induced by D-galactose (GalR/GalX) and also L-arabinose (AraR) and D-xylose (XlnR) in *A*. *nidulans* [33]. The expression of *hxkA* that encodes hexokinase involved in the last step of the oxido-reductive D-galactose catabolic pathway is not significantly reduced by any of the tested regulatory mutants (Fig 7b). Considering that the main role of *hxkA* is linked to glycolysis, it is likely to have a constitutive level of expression.

It should be noted that the ΔAΔGΔX strain is still able to grow on galactitol (Fig 4). We have three possible explanations for that: a) AraR, GalR and XlnR are not the only regulators involved in the D-galactose oxido-reductive pathway; b) the genes remain expressed at a basal, non-regulated level; c) there is an alternative pathway involved. A previous study suggested a pathway in which L-sorbose is phosphorylated, either directly or after epimerization to D-tagatose, and subsequently cleaved into two glycolytic triose-phosphates [16,17]. It should also be considered that *A*. *nidulans* possesses two D-galactose related regulators, GalR (tested in this study) and GalX. It is possible that GalX is directly involved in regulation of other enzymes of D-galactose conversion pathways. Future studies in which double and triple mutants of GalX with AraR and XlnR are analyzed will likely reveal the role of GalX in more detail.

## Conclusions

**Fig 8:**
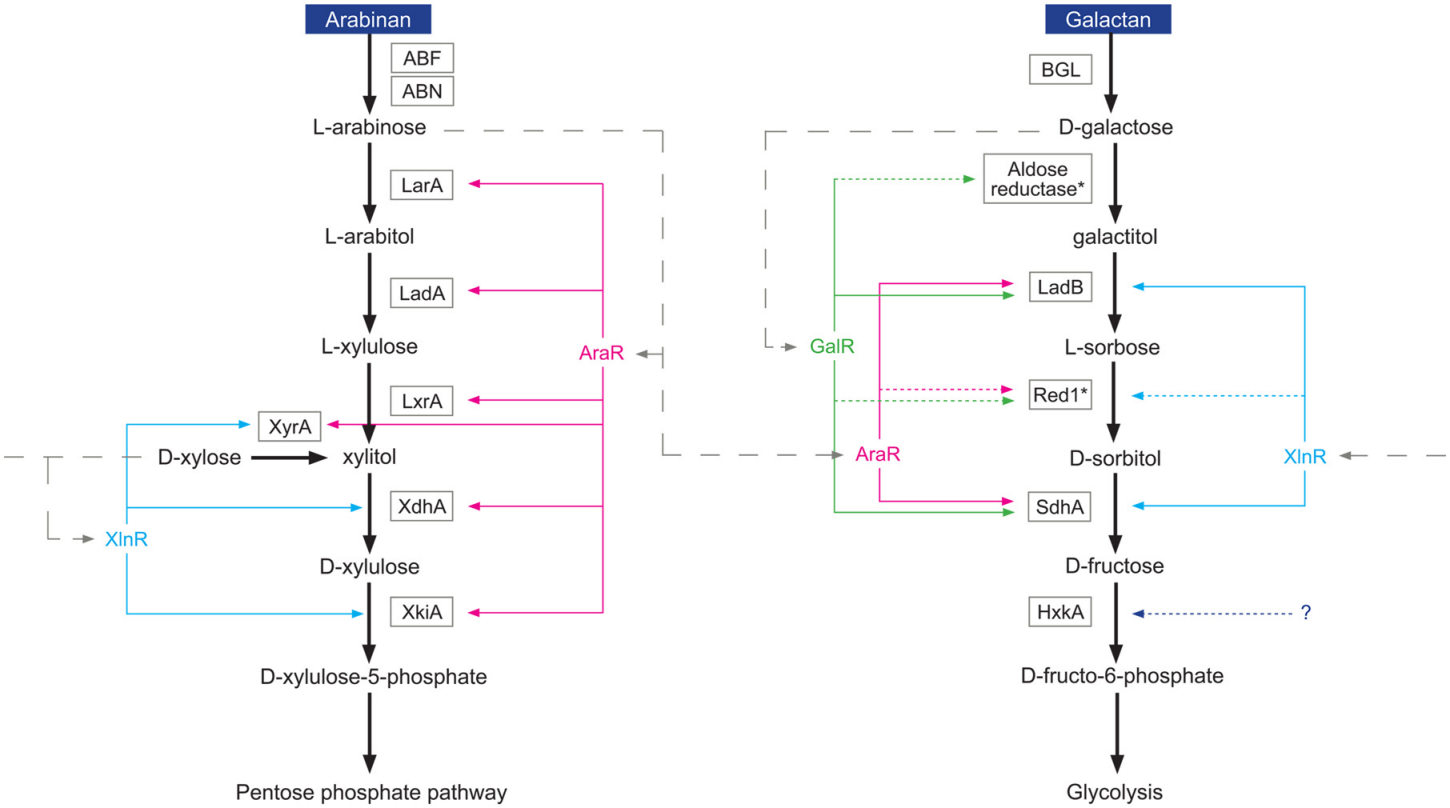
Regulation of the oxido-reductive D-galactose catabolic pathway and the pentose catabolic pathway (PCP) in *A*. *nidulans*. Genes of the PCP are regulated by the transcriptional activators XlnR (blue lines) and AraR (pink lines). Some of the D-galactose oxido-reductive genes are under regulatory control of GalR (green lines) together with XlnR and AraR. The expression of the hexokinase gene (*hxkA*) is observed in the *A*. *nidulans* ΔΔΔ triple mutant strain, probably due to the fact that this enzyme is also a part of glycolysis and therefore constitutively expressed. Some of the enzymatic conversions of the D-galactose oxido-reductive and the PCP pathway are verified, whereas others still need to be verified (marked by a star). Regulation of genes represented by solid lines is confirmed by expression analysis; dotted lines indicate that this regulation still needs to be verified. Dashed lines represent direct or indirect induction of TFs by monomer. Ortholog of *ladB*: AN4336, ortholog of *red1*: AN7914, ortholog of *sdhA*: AN2666.

To summarize, the data of this study demonstrate that regulation of carbon catabolic pathways requires co-operation of two or more transcriptional factors. We have presented evidence that the D-galactose oxido-reductive pathway is co-regulated by at least three TFs in *A*. *nidulans* (Fig 8). We have also observed a compensation effect, when in case of loss of one regulator the other takes over to maintain expression of necessary enzymes. Such a regulatory system by which metabolic pathway genes are co-regulated by multiple transcription factors especially benefits this cosmopolitan fungus that is known for its ability to quickly adapt to environmental changes. The common co-existence of D-galactose, L-arabinose and D-xylose in nature likely stimulated the evolution of an interactive regulatory network in which expression of genes is co-regulated by two or more TFs. It seems that this “strategy” has paid off since *A*. *nidulans* can degrade arabinogalactan, which is a combination of D-galactose and L-arabinose, better than *A*. *niger*. Recent study showed that *A*. *niger* failed to grow on galactose due to the absence of inducer uptake during germination [34]. In contrast, *A*. *nidulans* as well as *A*. *sydowii* and *A*. *versicolor* can use D-galactose as a sole carbon source (www.fung-growth.org). GalR, which is present in these species and absent in *A*. *niger*, may be required for galactose transporter expression during germination. Moreover, in this work we found evidence that *A*. *nidulans* has different enzymes involved in the PCP and D-galactose pathways, like was observed for *A*. *niger* [23]. However, two of the enzymes involved in the D-galactose oxido-reductive pathway in *A*. *nidulans* differ from those of *A*. *niger* and still remain to be characterized.

## Supporting Information

S1 Fig Pairwise amino acid sequence alignment of *A*. *nidulans* XlnR-AraR (a), GalR-AraR (b) and GalR-XlnR (c). The alignment was performed using Clustal Omega (http://www.ebi.ac.uk/Tools/msa/clustalo) [31] and visualized using Easy Sequencing in PostScript (http://espript.ibcp.fr/ESPript/ESPript/index.php) [32]. Conserved regions are marked by shaded boxes and similar regions by unshaded boxes. The Zn_2_Cys_6_ binuclear DNA binding domain is underlined and the six conserved cysteine residues are indicated by stars.
(PDF)

S2 Fig Synteny of the *galX* region among several *Aspergillus* species. The *galX* gene is conserved while the *galR* gene is present only in *A*. *nidulans*, *A*. *versicolor* and *A*. *sydowii*. The positions of *galX*, *galR*, *ladB* and *xhrA* are indicated.
(PDF)
