# Supplementary material for: Genetic Interaction of Aspergillus nidulans galR, xlnR and araR in Regulating D-Galactose and L-Arabinose Release and Catabolism Gene Expression
Source: PLoS One. 2015 Nov 18;10(11):e0143200. doi: 10.1371/journal.pone.0143200 (PMC4651341; doi:10.1371/journal.pone.0143200)
Supplement: S1 Fig — The alignment was performed using Clustal Omega (http://www.ebi.ac.uk/Tools/msa/clustalo) [31] and visualized using Easy Sequencing in PostScript (http://espript.ibcp.fr/ESPript/ESPript/index.php) [32]. Conserved regions are marked by shaded boxes and similar regions by unshaded boxes. The Zn2Cys6 binuclear DNA binding domain is underlined and the six conserved cysteine residues are indicated by stars. (PDF) [file pone.0143200.s001.pdf]

a)

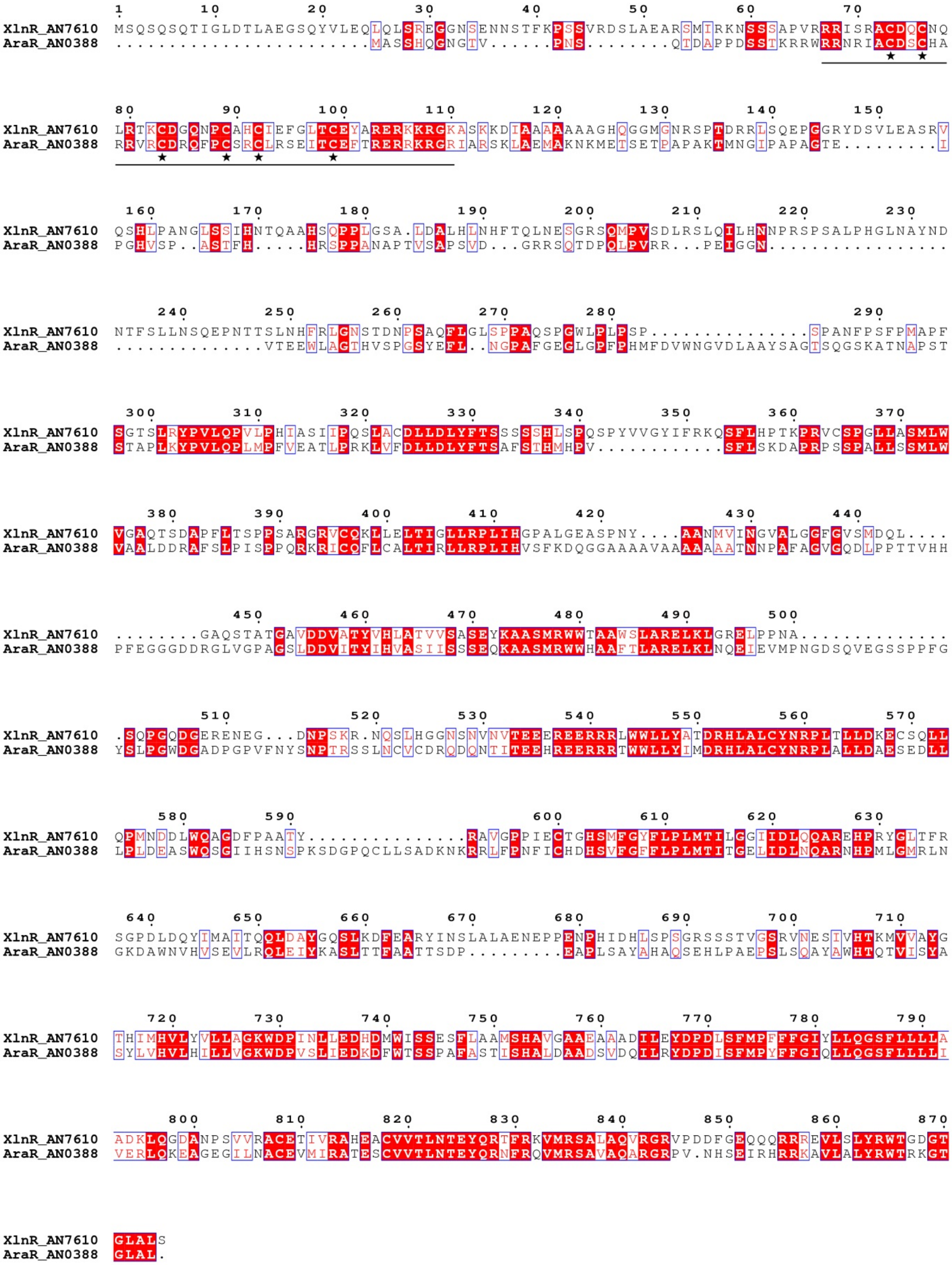
XlnR_AN7610 MSQSQSQTIGLDTLAEGSQYVLEQLQLSREGGNSENNSTFKPSSVRDSLAEARSMIRKNSSSAPVRRRISRACDQCNQ
AraR_AN0388 .......................MASSHQGNGTV......PNS........QTDAPPDSSTKRRWRRNRIACDSCHA
XlnR_AN7610 LRTKCDGQNPCAHCIEFGLTCEYARERKKRGKASKKDIAAAAAAGHQGGMGNRSPTDRRLSQEPGGRYDSVLEASRV
AraR_AN0388 RRVRCDRQFPCSRCLRSEITCEFTRERRKRGRIARSKLAEMAKNKMETSETPAPAKTMNGIPAPAGTE.........I
XlnR_AN7610 QSHLPANGLSSIHNTQAAHSQPPLGSA.LDALHLNHFTQLNESGRSQMPVSDLRSLQILHNNPRSPSALPHGLNAYND
AraR_AN0388 PGHVSP..ASTFH.....HRSPPANAPTVSAPSVD...GRRSQTDPQLPVRR...PEIGGN.................
XlnR_AN7610 NTFSLLNSQEPNTTSLNHFRLGNSTDNPSAQFLGLSPPAQSPGWLPLPSP...............SPANFPSFPMAPF
AraR_AN0388 ..............VTEEWLAGTHVSPGSYEFL..NGPAFGEGLGPFPHMFDVWNGVDLAAYSAGTSQGSKATNAPST
XlnR_AN7610 SGTSLRYPVLQPVLPHIASIIPQSLACDLLDLYFTSSSSSHLSPQSPYVVGYIFRKQSFLHPTKPRVCSPGLLASMLW
AraR_AN0388 STAPLKYPVLQPLMPFVEATLPRKLVFDLLDLYFTSAFSTHMHPV............SFLSKDAPRPSSPALLSSMLW
XlnR_AN7610 VGAQTSDAPFLTSPPSARGRVCQKLLELTIGLLRPLIHGPALGEASPNY....AANMVINGVALGGFGVSMDQL....
AraR_AN0388 VAALDDRAFSLPISPPQRKRICQFLCALTIRLLRPLIHVSFKDQGGAAAAVAAAAAAATNNPAFAGVGQDLPPTTVHH
XlnR_AN7610 ........GAQSTATGAVDDVATYVHLATVVSASEYKAASMRWWTAAWSLARELKLGRELPPNA..............
AraR_AN0388 PFEGGGDDRGLVGPAGSLDDVITYIHVASIISSSEQKAASMRWWHAAFTLARELKLNQEIEVMPNGDSQVEGSSPPFG
XlnR_AN7610 .SQPGQDGERENEG...DNPSKR.NQSLHGGNSNVNVTEEEREERRRLWWLLYATDRHLALCYNRPLTLLDKECSQLL
AraR_AN0388 YSLPGWDGADPGPVFNYSNPTRSSLNCVCDRQDQNTITEEHREERRRTWWLLYIMDRHLALCYNRPLALLDAESEDLL
XlnR_AN7610 QPMNDDLWQAGDFPAATY...............RAVGPPIECTGHSMFGYFLPLMTILGGIIDLQQAREHPRYGLTFR
AraR_AN0388 LPLDEASWQSGIIHSNSPKSDGPQCLLSADKNKRRLFPNFICHDHSVFGFFLPLMTITGELIDLNQARNHPMLGMRLN
XlnR_AN7610 SGPDLDQYIMAITQQLDAYGQSLKDFEARYINSLALAENEPPENPHIDHLSPSGRSSSTVGSRVNESIVHTKMVVAYG
AraR_AN0388 GKDAWNVHVSEVLRQLEIYKASLTTFAATTSDP.........EAPLSAYAHAQSEHLPAEPSLSQAYAWHTQTVISYA
XlnR_AN7610 THIMHVLYVLLAGKWDPINLLEDHDMWISSESFLAAMSHAVGAAEAAADILEYDPDLSFMPFFFGIYLLQGSFLLLLA
AraR_AN0388 SYLVHVLHILLVGKWDPVSLIEDKDFWTSSPAFASTISHALDAADSVDQILRYDPDISFMPYFFGIQLLQGSFLLLLI
XlnR_AN7610 ADKLQGDANPSVVRACETIVRAHEACVVTLNTEYQRTFRKVMRSALAQVRGRVPDDFGEQQQRRREVLSLYRWTGDGT
AraR_AN0388 VERLQKEAGEGILNACEVMIRATESCVVTLNTEYQRNFRQVMRSAVAQARGRPV.NHSEIRHRRKAVLALYRWTRKGT
XlnR_AN7610 GLALS
AraR_AN0388 GLAL.

b)

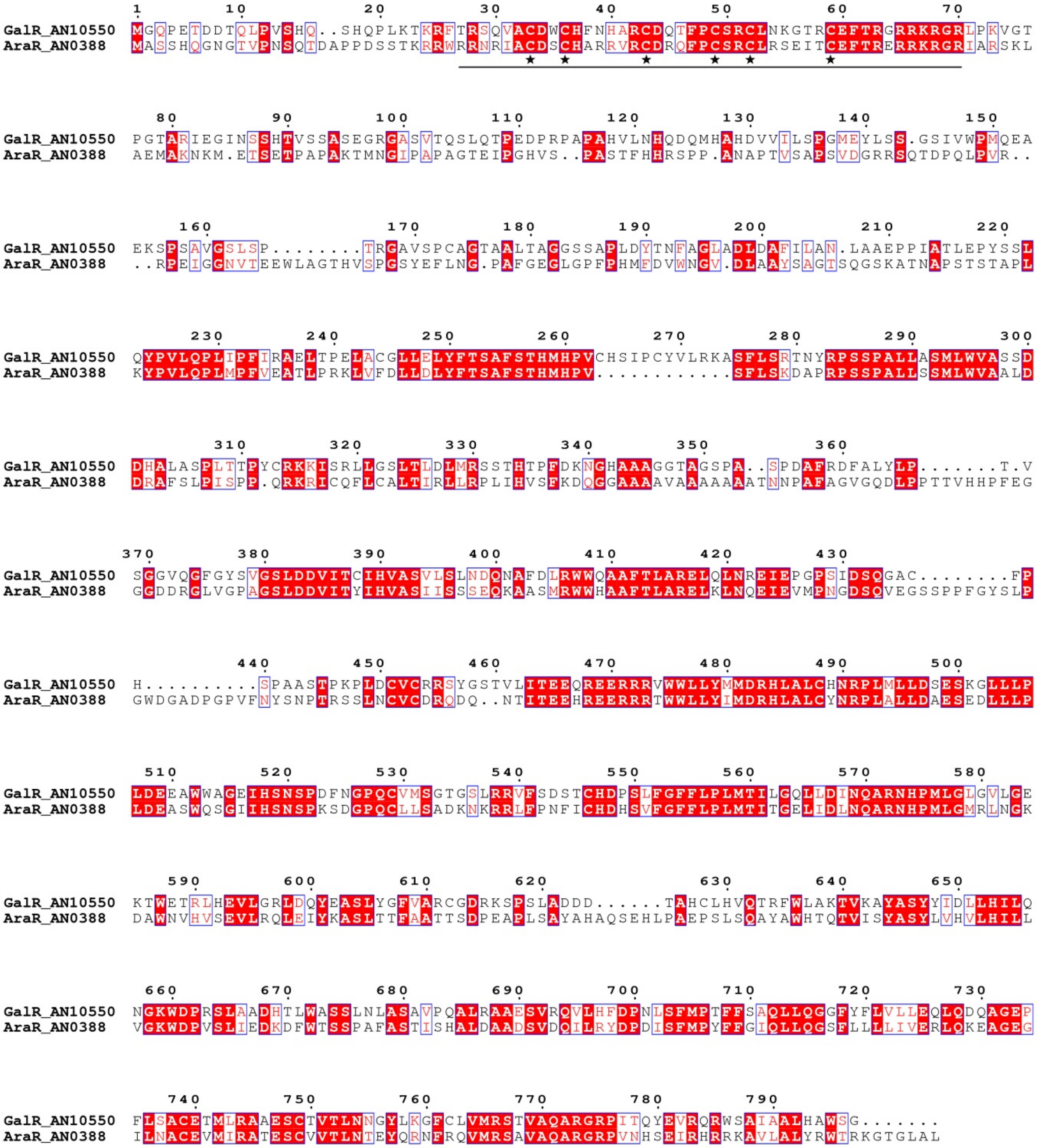

GalR_AN10550 MGQPETDDTQLPVSHQ..SHQPLKTKRFTRSQVACDWCHFNHARCDQTFPCSRCLNKGTRCEFTRGRRKRGRLPKVGT
AraR_AN0388 MASSHQGNGTVPNSQTDAPPDSSTKRRWRRNRIACDSCHARRVRCDRQFPCSRCLRSEITCEFTRERRKRGRIARSKL
GalR_AN10550 PGTARIEGINSSHTVSSASEGRGASVTQSLQTPEDPRPAPAHVLNHQDQMHAHDVVILSPGMEYLSS.GSIVWPMQEA
AraR_AN0388 AEMAKNKM.ETSETPAPAKTMNGIPAPAGTEIPGHVS..PASTFHHRSPP.ANAPTVSAPSVDGRRSQTDPQLPVR..
GalR_AN10550 EKSPSAVGSLSP........TRGAVSPCAGTAALTAGGSSAPLDYTNFAGLADLDAFILAN.LAAEPPIATLEPYSSL
AraR_AN0388 ..RPEIGGNVTEEWLAGTHVSPGSYEFLNG.PAFGEGLGPFPHMFDVWNGV.DLAAYSAGTSQGSKATNAPSTSTAPL
GalR_AN10550 QYPVLQPLIPFIRAELTPELACGLLELYFTSAFSTHMHPVCHSIPCYVLRKASFLSRTNYRPSSPALLASMLWVASSD
AraR_AN0388 KYPVLQPLMPFVEATLPRKLVFDLLDLYFTSAFSTHMHPV...........SFLSKDAPRPSSPALLSSMLWVAALD
GalR_AN10550 DHALASPLTTPYCRKKISRLLGSLTLDLMRSSTHTPFDKNGHAAAGGTAGSPA..SPDAFRDFALYLP.......T.V
AraR_AN0388 DRAFSLPISPP.QRKRICQFLCALTIRLLRPLIHVSFKDQGGAAAAVAAAAAATNNPAFAGVGQDLPPTTVHHPFEG
GalR_AN10550 SGGVQGFGYSVGSLDDVITCIHVASVLSLNDQNAFDLRWWQAAFTLARELQLNREIEPGPSIDSQGAC........FP
AraR_AN0388 GGDDRGLVGPAGSLDDVITYIHVASIISSSEQKAASMRWWHAAFTLARELKLNQEIEVMPNGDSQVEGSSPPFGYSLP
GalR_AN10550 H..........SPAASTPKPLDCVCRRSYGSTVLITEEQREERRRVWWLLYMMDRHLALCHNRPLMLLDSESKGLLLP
AraR_AN0388 GWDGADPGPVFNYSNPTRSSLNCVCDRQDQ..NTITEEHREERRRTWWLLYIMDRHLALCYNRPLALLDAESEDLLLP
GalR_AN10550 LDEEAWWAGEIHSNSPDFNGPQCVMSGTGSLRRVFSDSTCHDPSLFGFFLPLMTILGQLLDINQARNHPMLGLGVLGE
AraR_AN0388 LDEASWQSGIIHSNSPKSDGPQCLLSADKNKRRLFPNFICHDHSVFGFFLPLMTITGELIDLNQARNHPMLGMRLNGK
GalR_AN10550 KTWETRLHEVLGRLDQYEASLYGFVARCGDRKSPSLADDD......TAHCLHVQTRFWLAKTVKAYASYYIDLLHILQ
AraR_AN0388 DAWNVHVSEVLRQLEIYKASLTTFAATTSDPEAPLSAYAHAQSEHLPAEPSLSQAYAWHTQTVISYASYLVHVLHILL
GalR_AN10550 NGKWDPRSLAADHTLWASSLNLASAVPQAIRAAESVRQVLHFDPNLSFMPTFFSAQLLQGGFYFLVLLEQLQDQAGEP
AraR_AN0388 VGKWDPVSLIEDKDFWTSSPAFASTISHALDAADSVDQILRYDPDISFMPYFFGIQLLQGSFLLLLIVERLQKEAGEG
GalR_AN10550 FLSACETMLRAAESCTVTLNNGYLKGFCLVMRSTVAQARGRPITQYEVRQRWSAIAALHAWSG.......
AraR_AN0388 ILNACEVMIRATESCVVTLNTEYQRNFRQVMRSAVAQARGRPVNHSEIRHRRKAVLALYRWTRKGTGLAL

c)

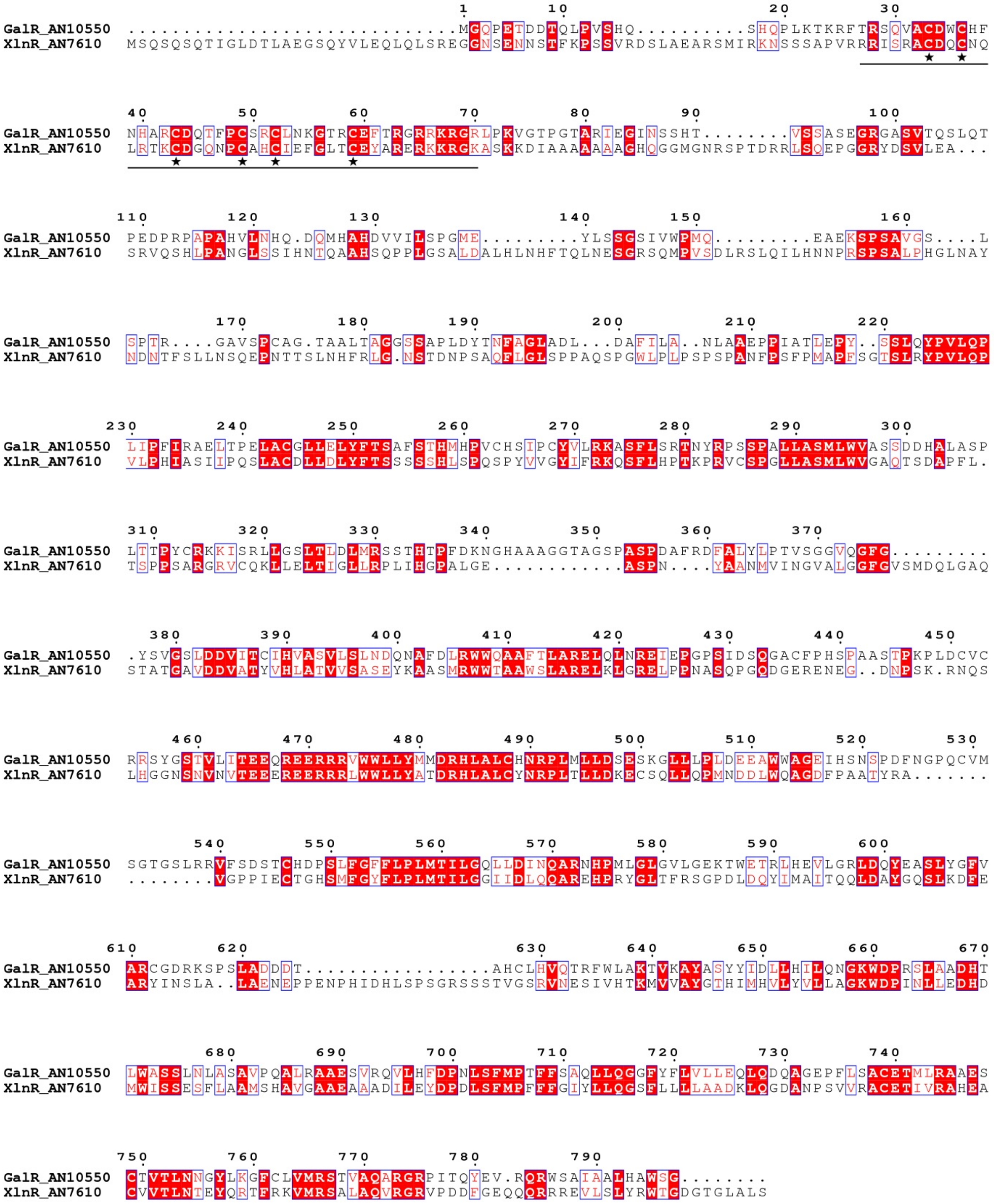

1 10 20 30
GalR_AN10550 ..............................MGQPETDDTQLPVSHQ..........SHQPLKTKRFTRSQVACDWCHF
XlnR_AN7610 MSQSQSQTIGLDTLAEGSQYVLEQLQLSREGGNSENNSTFKPSSVRDSLAEARSMIRKNSSSAPVRRRISRACDQCNQ
40 50 60 70 80 90 100
GalR_AN10550 NHARCDQTFPCSRCLNKGTRCEFTRGRRKRGRLPKVGTPGTARIEGINSSHT........VSSASEGRGASVTQSLQT
XlnR_AN7610 LRTKCDGQNPCAHCIEFGLTCEYARERKKRGKASKKDIAAAAAAAGHQGGMGNRSPTDRRLSQEPGGRYDSVLEA...
110 120 130 140 150 160
GalR_AN10550 PEDPRPAPAHVLNHQ.DQMHAHDVVILSPGME.........YLSSGSIVWPMQ.........EAEKSPSAVGS....L
XlnR_AN7610 SRVQSHLPANGLSSIHNTQAAHSQPPLGSALDALHLNHFTQLNESGRSQMPVSDLRSLQILHNNPRSPSALPHGLNAY
170 180 190 200 210 220
GalR_AN10550 SPTR....GAVSPCAG.TAALTAGGSSAPLDYTNFAGLADL...DAFILA..NLAAEPPIATLEPY..SSLQYPVLQP
XlnR_AN7610 NDNTFSLLNSQEPNTTSLNHFRLG.NSTDNPSAQFLGLSPPAQSPGWLPLPSPSPANFPSFPMAPFSGTSLRYPVLQP
230 240 250 260 270 280 290 300
GalR_AN10550 LIPFIRAELTPELACGLLELYFTSAFSTHMHPVCHSIPCYVLRKASFLSRTNYRPSSPALLASMLWVASSDDHALASP
XlnR_AN7610 VLPHIASIIPQSLACDLLDLYFTSSSSSHLSPQSPYVVGYIFRKQSFLHPTKPRVCSPGLLASMLWVGAQTSDAPFL.
310 320 330 340 350 360 370
GalR_AN10550 LTTPYCRKKISRLLGSLTLDLMRSSTHTPFDKNGHAAAGGTAGSPASPDAFRDFALYLPTVSGGVQGFG.........
XlnR_AN7610 TSPPSARGRVCQKLLELTIGLLRPLIHGPALGE............ASPN....YAANMVINGVALGGFGVSMDQLGAQ
380 390 400 410 420 430 440 450
GalR_AN10550 .YSVGSLDDVITCIHVASVLSLNDQNAFDLRWWQAAFTLARELQLNREIEPGPSIDSQGACFPHSPAASTPKPLDCVC
XlnR_AN7610 STATGAVDDVATYVHLATVVSASEYKAASMRWWTAAWSLARELKLGRELPPNASQPGQDGERENEG..DNPSK.RNQS
460 470 480 490 500 510 520 530
GalR_AN10550 RRSYGSTVLITEEQREERRRVWWLLYMMDRHLALCHNRPLMLLDSESKGLLLPLDEEAWWAGEIHSNSPDFNGPQCVM
XlnR_AN7610 LHGGNSNVNVTEEEREERRRLWWLLYATDRHLALCYNRPLTLLDKECSQLLQPMNDDLWQAGDFPAATYRA.......
540 550 560 570 580 590 600
GalR_AN10550 SGTGSLRRVFSDSTCHDPSLFGFFLPLMTILGQLLDINQARNHPMLGLGVLGEKTWETRLHEVLGRLDQYEASLYGFV
XlnR_AN7610 ........VGPPIECTGHSMFGYFLPLMTILGGIIDLQQAREHPRYGLTFRSGPDLDQYIMAITQQLDAYGQSLKDFE
610 620 630 640 650 660 670
GalR_AN10550 ARCGDRKSPSLADDDT.................AHCLHVQTRFWLAKTVKAYASYYIDLLHILQNGKWDPRSLAADHT
XlnR_AN7610 ARYINSLA..LAENEPPENPHIDHLSPSGRSSSTVGSRVNESIVHTKMVVAYGTHIMHVLYVLLAGKWDPINLLEDHD
680 690 700 710 720 730 740
GalR_AN10550 LWASSLNLASAVPQALRAAESVRQVLHFDPNLSFMPTFFSAQLLQGGFYFLVLLEQLQDQAGEPFLSACETMLRAAES
XlnR_AN7610 MWISSESFLAAMSHAVGAAEAAADILEYDPDLSFMPFFFGIYLLQGSFLLLLAADKLQGDANPSVVRACETIVRAHEA
750 760 770 780 790
GalR_AN10550 CTVTLNNGYLKGFCLVMRSTVAQARGRPITQYEV.RQRWSAIAALHAWSG........
XlnR_AN7610 CVVTLNTEYQRTFRKVMRSALAQVRGRVPDDFGEQQQRRREVLSLYRWTGDGTGLALS
