# Supplementary figures and images for: Genetic Interaction of Aspergillus nidulans galR, xlnR and araR in Regulating D-Galactose and L-Arabinose Release and Catabolism Gene Expression
Source: PLoS One. 2015 Nov 18;10(11):e0143200. doi: 10.1371/journal.pone.0143200 (PMC4651341; doi:10.1371/journal.pone.0143200)

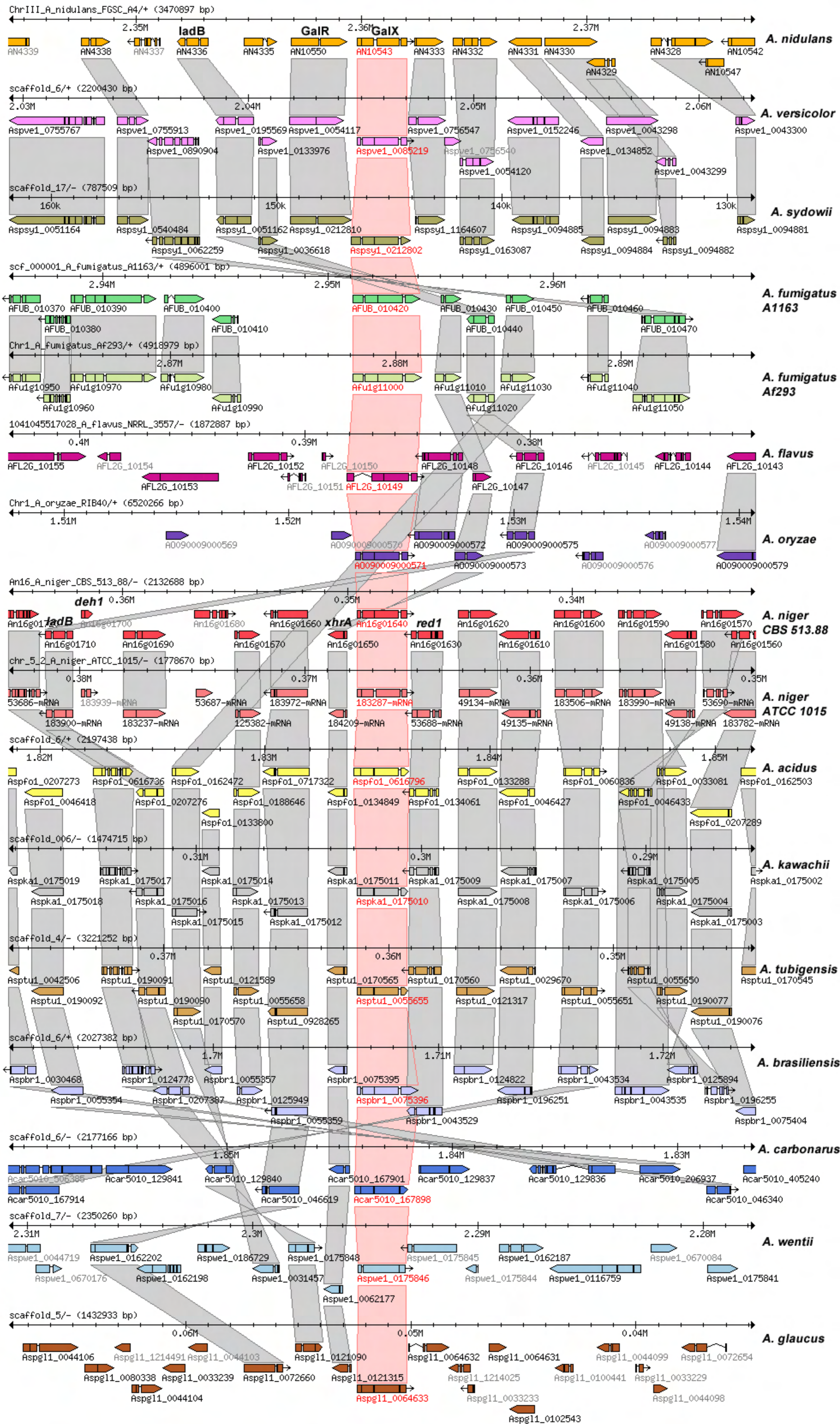

Supplement: S2 Fig — The galX gene is conserved while the galR gene is present only in A. nidulans, A. versicolor and A. sydowii. The positions of galX, galR, ladB and xhrA are indicated. (PDF) [file pone.0143200.s002.pdf]
